# The role of histologic subtype, p16^INK4a^ expression, and presence of human papillomavirus DNA in penile squamous cell carcinoma

**DOI:** 10.1186/s12885-015-1268-z

**Published:** 2015-04-03

**Authors:** Julie Steinestel, Andreas Al Ghazal, Annette Arndt, Thomas J Schnoeller, Andres J Schrader, Peter Moeller, Konrad Steinestel

**Affiliations:** 1 Department of Urology, Muenster University Medical Center, Albert-Schweitzer-Campus 1, A1, 48149 Muenster, Germany; 2 Department of Urology, University of Ulm, Prittwitzstrasse 43, 89075 Ulm, Germany; 3 Institute of Pathology and Molecular Pathology, Bundeswehrkrankenhaus Ulm, Oberer Eselsberg 40, 89081 Ulm, Germany; 4 Institute of Pathology, University of Ulm, Albert-Einstein-Allee 23, 89070 Ulm, Germany; 5 Gerhard Domagk Institute of Pathology, University of Muenster, Domagkstrasse 17, 48149 Muenster, Germany

**Keywords:** Human papillomavirus, Invasion, Metastasis, p16^INK4a^, Penile cancer

## Abstract

**Background:**

Up to 50% of penile squamous cell carcinomas (pSCC) develop in the context of high-risk human papillomavirus (HR-HPV) infection. Most of these tumours have been reported to show basaloid differentiation and overexpression of tumour suppressor protein p16^INK4a^. Whether HPV-triggered carcinogenesis in pSCC has an impact on tumour aggressiveness, however, is still subject to research.

**Methods:**

In tissue specimens from 58 patients with surgically treated pSCC between 1995 and 2012, we performed p16^INK4a^ immunohistochemistry and DNA extraction followed by HPV subtyping using a PCR-based approach. The results were correlated with histopathological and clinical parameters.

**Results:**

90.4% of tumours were of conventional (keratinizing) subtype. HR-HPV DNA was detected in 29.3%, and a variety of p16^INK4a^ staining patterns was observed in 58.6% of samples regardless of histologic subtype. Sensitivity of basaloid subtype to predict HR-HPV positivity was poor (11.8%). In contrast, sensitivity and specificity of p16^INK4a^ staining to predict presence of HR-HPV DNA was 100% and 57%, respectively. By focussing on those samples with intense nuclear staining pattern for p16^INK4a^, specificity could be improved to 83%. Both expression of p16^INK4a^ and presence of HR-HPV DNA, but not histologic grade, were inversely associated with pSCC tumour invasion (p = 0.01, p = 0.03, and p = 0.71). However, none of these correlated with nodal involvement or distant metastasis. In contrast to pathological tumour stage, the HR-HPV status, histologic grade, and p16^INK4a^ positivity failed to predict cancer-specific survival.

**Conclusions:**

Our results confirm intense nuclear positivity for p16^INK4a^, rather than histologic subtype, as a good predictor for presence of HR-HPV DNA in pSCC. HR-HPV / p16^INK4a^ positivity, independent of histological tumour grade, indicates a less aggressive local behaviour; however, its value as an independent prognostic indicator remains to be determined. Since local invasion can be judged without p16^INK4a^/HPV-detection on microscopic evaluation, our study argues against routine testing in the setting of pSCC.

## Background

Squamous cell carcinoma of the penis (pSCC) is associated with high morbidity and mortality and causes severe psychological impact [1,2]. High-risk human papillomavirus (HR-HPV) DNA is detectable in 30-80% of pSCC surgical specimens [3]. In total, more than 17,000 cancer cases in European men per year are attributable to HPV, with more than 15,000 of them being specifically attributable to HR-HPV-16 or -18 [4]. The benefit of early vaccination of girls and boys using a quadrivalent HPV vaccine to prevent later occurrence of HPV-related diseases is a current subject of discussion [5]. Integration of HR-HPV DNA into the host cell genome leads to overexpression of viral oncoproteins E6 and E7 that exert a dysregulating effect on cell cycle control [6]; upregulation of tumour suppressor protein p16^INK4a^ is understood as an attempt to stop uncontrolled cell proliferation in response to HPV infection [7]. P16^INK4a^ immunohistochemistry is therefore used as a surrogate marker for high-risk HPV in cancers of the cervix uteri and the head and neck region [8,9]. In bladder cancer, however, we did not detect HPV DNA in 29 samples of urothelial carcinoma *in situ* despite diffuse intense confluent staining pattern for p16^INK4a^ [10]. Chaux et al. reported that in pSCC, HPV-associated tumours are frequently composed of undifferentiated warty or basaloid cells that show a variable degree of koilocytic changes. The same group also noted that non-HPV tumours are composed of keratinizing differentiated squamous cells [11]. Concerning prognosis, HPV-driven squamous cell carcinomas of the head and neck region (HNSCCs) are associated with significantly improved progression-free survival and disease-free survival [12]. On the other hand, published data suggests a role for HPV-16 E6/E7 oncoproteins in the induction of epithelial-mesenchymal transition which may contribute to cell migration and metastasis [13,14]. Therefore, the impact of virus-associated tumourigenesis on tumour aggressiveness, metastatic potential, and patient prognosis in penile cancer is still unclear, since only a few studies have addressed this topic, and the results are inconsistent [15-17]. In the present study, we analysed pSCC tissue samples for expression of p16^INK4a^ and presence of HPV DNA and correlated the results with tumour- and patient-specific characteristics as well as cancer-specific survival. We evaluated the value of histologic subtype and various p16^INK4a^ staining patterns as indicators for the presence of HR-HPV DNA in tumour tissue, and showed for the first time that presence of HR-HPV DNA in pSCC is inversely associated with local tumour invasion. Furthermore, we analysed the impact of HPV-driven tumourigenesis on cancer-specific survival in pSCC patients.

## Methods

### Ethics statement

All specimens were surgically removed for therapeutic purposes and subsequent histologic examination and were thoroughly pseudonymized for the use in this study. Therefore, individual written informed consent was not mandatory. The study was approved by the University of Ulm ethics committee (Approval No. 331/2013).

### Patient samples

Tissue samples from 58 patients were included in the study; for all patients, biological parameters and tumour characteristics were available. Patients underwent surgery for penile cancer between 1995 and 2012 at the Ulm University Medical Centre (n = 37) or at the Bundeswehrkrankenhaus Ulm (n = 21). All specimens had been submitted for routine histologic examination to the Institutes of Pathology of either the University of Ulm or the Bundeswehrkrankenhaus Ulm. Clinico-pathological characteristics are summarized in Table 1. TNM stages were determined according to the UICC classification of 2010 [18]. T stages pTis (carcinoma in situ) and pTa (papillary/verrucous carcinoma) were summarized as noninvasive, while pT1-pT4 stages were classified as invasive lesions. Patient and tumour characteristics obtained from the institutional databases included age, stage, and the presence of histologically verified regional lymph node involvement or distant metastasis, respectively. Of 35 patients (60.3% of the complete cohort; 23 HPV-negative and 12 HPV-positive cases), complete follow-up data was obtainable and reached up to 204 months (median/mean follow-up 15/31 months).

**Table 1:** **Clinico-pathologic sample characteristics**

No. of patients	58		
**No. of samples**	58		
**Age (mean; range)**	64.5; 31-93 years		
**Tumour characteristics**			
**Differentiation**		**Distant metastasis**	
keratinizing	53	Mx	21
basaloid	5	cM0	33
**Grading**		pM+	4
Low-grade (G1-2)	43		
High-grade (G3-4)	15	**p16** ^**INK4a**^ **IHC**	
**pT stage**		negative	24
pTis	10	positive	34
pTa	2	intense confluent	24
pT1	19	focally scattered	10
pT2	20		
pT3	5		
pT4	2	**HPV subtyping**	
**Nodal involvement**		HPV-	40
Nx	30	HPV+	18
pN0	15	HPV-16	16
pN+	13	HPV-45	1
		HPV-6	1

pTis, carcinoma in situ; pTa, papillary/exophytic (noninvasive) carcinoma; pT1-pT4, depth of tumor invasion; Nx, lymph node status unknown; pN0, (verified) absence of lymph node metastases; pN+, (verified) presence of lymph node metastases; Mx, presence of distant metastases unknown; cM0, no clinical evidence for distant metastases; pM+, (verified) presence of distant metastases; IHC, immunohistochemistry; HPV, human papillomavirus.

### Immunohistochemistry, image acquisition, and expression analysis

Immunohistochemistry for p16^INK4a^ was performed on a BenchMark Autostainer (Ventana Medical Systems, Tucson, USA) according to the manufacturer’s protocol using a prediluted mouse monoclonal antibody (CINtec® p16 Histology, clone E6H4, Ventana Medical Systems, Tucson, USA). Microscopic slide evaluation/image acquisition was performed using a Leica DM6000B light microscope (Leica, Wetzlar, Germany) and the Diskus Mikroskopische Diskussion image acquisition software (Carl H. Hilgers, Königswinter, Germany). P16^INK4a^ staining patterns were classified in the following categories: a) intense confluent or b) focally scattered nuclear and/or cytoplasmic staining pattern [19]. Histological subtype, tumour grade and p16^INK4a^ staining patterns were confirmed by an experienced pathologist (PM).

### Human papillomavirus (HPV) testing

**Figure 1:**
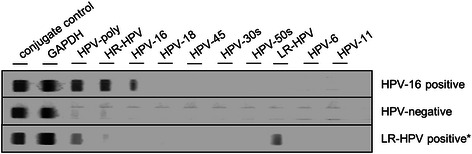
**Representative results of HPV genotyping using a PCR-based approach.** First lane shows a HPV-16-positive case, while in lane 2, no HPV DNA was detectable. Lane 3 shows detection of LR-HPV DNA that was later identified as HPV isoform 6. *LR-HPV, low-risk human papillomavirus.*

HPV genotyping was performed as previously described [10]. In short, DNA was extracted from tumour-containing paraffin slides using the automated Maxwell® 16 FFPE Plus LEV DNA Purification Kit (Promega, Madison, USA) and amplified with biotinylated primers; PCR products were incubated with oligonucleotide-precoated strips using the HPV typing kit from AID diagnostics (Strassberg, Germany) according to the manufacturer’s protocol (Figure 1). The kit detects 15 different HPV genotypes (6, 11, 16, 18, 31, 33, 35, 39, 45, 51, 52, 53, 56, 58 and 59).

### Statistical analysis

Correlations between dichotomous variables (histologic subtype, p16^INK4a^ positivity, HPV status, presence/absence of tumour invasion, presence/absence of lymph node involvement or distant metastasis, tumour differentiation grade) were tested for significance using the Chi-Square/Fisher’s exact test (SPSS, IBM, Ehningen, Germany). The difference in disease-specific survival between low-grade/high-grade, p16^INK4a^+/p16^INK4a^- and HPV+/HPV- groups was assessed using LogRank test (SPSS, IBM, Ehningen, Germany). A two-tailed *p*-value of <0.05 was regarded as statistically significant.

## Results

### Clinico-pathologic sample characteristics

**Figure 2:**
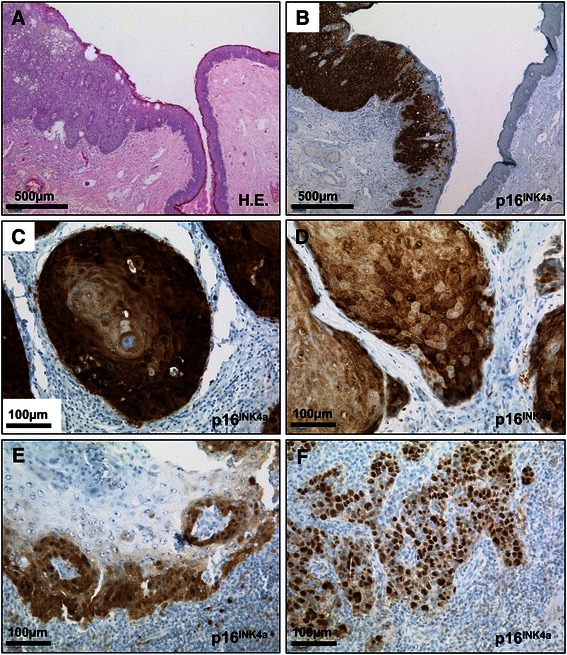
**Representative microphotographs of pSCC specimens. A** and **B**, penile squamous cell carcinoma *in situ* displaying strong, confluent expression of p16^INK4a^. Note ascending p16^INK4a^ positivity in non-malignant epithelium adjacent to carcinoma. **C-F**, variety of p16^INK4a^ staining patterns in pSCC: **C**, strong, confluent staining in invasive keratinizing carcinoma; **D**, diffuse and **E**, focally scattered positivity for p16^INK4a^; **F**, almost exclusively nuclear immunostaining. *Scale bars as indicated; H.E., haematoxylin-eosin; HR-HPV, high-risk human papillomavirus; pSCC, penile squamous cell carcinoma.*

The mean (median) age of the 58 patients included in the study was 64.5 (64.0) years (IQR, 55.5-73.3 years, Table 1). 43/58 (74.1%) samples were histologically classified as low-grade (G1-2) tumours; 53/58 (91.4%) of the specimens showed keratinizing growth pattern with formation of keratin pearls (Figure 2C), while 5/58 (8.6%) tumours were of the basaloid subtype. Non-invasive tumour growth (pTis/pTa stages) was observed in 12/58 (20.7%) samples. Presence of lymph node or distant metastases was confirmed in pathological workup in 13 (22.4%) and 4 (6.9%) cases, respectively.

### P16^INK4a^ Immunohistochemistry

Altogether, positive immunostaining for p16^INK4a^ was observed in 34 (58.6%) tumour samples, with 30/53 (56.6%) tumours of the keratinizing subtype showing some degree of positivity. However, there was a wide variety in p16^INK4a^ expression patterns (Figure 2): intense confluent staining pattern (Figure 2B and C) was detected in 24 of the 34 (70.6%) positive cases, while weak or focal scattered staining (Figure 2D and E) was observed in 10 (29.4%) samples. Among the cases with ubiquitous strong expression in all tumour cells, there was also a variety in cellular distribution of p16^INK4a^ from almost exclusively nuclear (3 cases, Figure 2F) to intense cytoplasmic and nuclear positivity (22 cases, Figure 2B and C). There was no significant association between the observed p16^INK4a^ staining pattern and the respective area of individual tumors (superficial/ exophytic versus invasive areas).

### HPV genotyping

Genomic subtyping following DNA extraction confirmed presence of HPV DNA in 18 (31.0%) of 58 samples, 16 of which were classified as HPV-16 (Figure 1). One sample turned out to harbour HPV-45, while one case was positive for HPV-6 DNA (low risk HPV). Notably, PCR amplification of HPV-DNA was also successful in one case where the paraffin block dated back to 1995.

### HPV status and clinic-pathological parameters

Basaloid histologic subtype of pSCC was not significantly associated with presence of HR-HPV (HPV-16 or HPV-45) in our study cohort (p = 0.62; Fisher’s exact test; Table 2). Although the specificity of the basaloid growth pattern for HR-HPV was 92.7%, since 28.3% of the keratinizing tumours were also found to be HR-HPV positive, sensitivity was only 11.8% (positive predictive value (PPV), 40%; Table 3). P16^INK4a^ immunopositivity, irrespective of the observed staining pattern, was significantly associated with presence of HR-HPV DNA (p < 0.001; Fisher’s exact test; Table 2). However, the PPV of p16^INK4a^ staining for presence of HR-HPV was only 52.9% (sensitivity, 100%; specificity, 60%). This predictor was markedly improved by focusing on cases with intense nuclear and confluent staining patterns in all tumour cells (PPV, 75%; specificity, 85%; Table 3). Histopathologic tumour grade was not significantly associated with presence of HPV DNA. HR-HPV positivity and nuclear p16^INK4a^ staining, but not histologic differentiation grade, was significantly associated with non-invasive tumour growth (pTis/pTa stage; p = 0.03, p = 0.01, and p = 0.71). For verified nodal involvement or distant metastasis, there was no significant association with HPV status, p16^INK4a^ positivity or grade of differentiation of the primary tumour (p = 0.22, p = 0.25, and p = 0.41; Fisher’s exact test).

**Table 2:** **HPV status and clinico-pathological parameters**

	HR-HPV-	HR-HPV+	*p*-value*
**Histopathologic subtype**			
keratinizing	38	15	
basaloid	3	2	**0.62**
**Grading**			
Low-grade (G1-2)	32	11	
High-grade (G3-4)	9	6	**0.33**
**Presence of tumour invasion**			
Non-invasive	5	7	
Invasive	36	10	**0.028**
**Presence of metastasis**			
N0M0	10	5	
N+ and/or M+	11	2	**0.40**
**p16** ^**INK4a**^ **immunostaining**			
negative	24	0	
scattered	10	0	
intense nuclear/confluent	7	17	**<0.001**

*Fisher’s exact Test.

**Table 3:** **Test statistics for basaloid histologic subtype and p16** ^**INK4a**^ **immunostaining to predict HR-HPV positivity in pSCC**

Test	ppV (%)	Sensitivity (%)	Specificity (%)
Basaloid histologic subtype	40	11	95
Any positivity for p16^INK4a^	53	100	60
Intense nuclear p16^INK4a^ staining	75	100	85

### HPV status and cancer-specific survival

**Figure 3:**
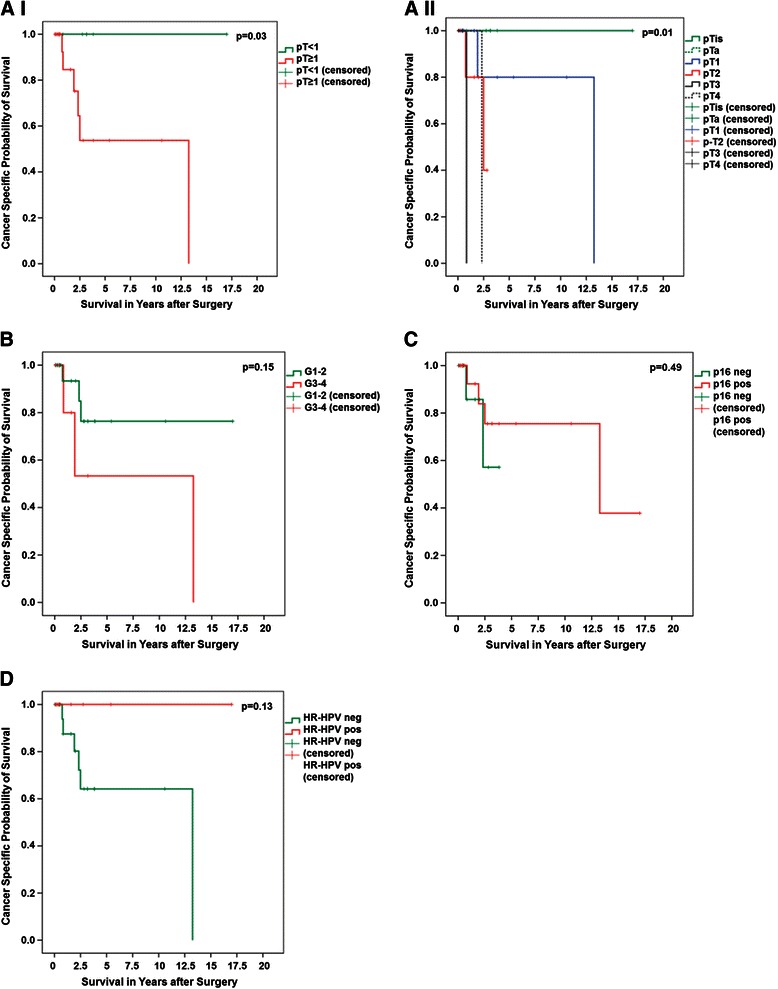
**Cancer-specific survival analysis. A-I and -II**, Cancer-specific survival depending on histopathologically confirmed T stage as indicated; **B**, depending on histopathologic differentiation grade; **C**, depending on p16^INK4a^ expression; **D**, depending on HR-HPV status. *G1-4, histopathologic differentiation grade; HR-HPV, high-risk human papillomavirus.*

Follow-up data was obtainable for 35 patients and reached up to 204 months. No patient in the HPV-positive group, but 6 patients in the HPV-negative group died from the disease (Figure 3). However, this difference failed to reach significance in Kaplan-Meier analysis (p = 0.13; Log Rank test). In contrast to pathological tumour stage including all stages (p = 0.01), p16^INK4a^ positivity and histologic differentiation grade (G1-2 vs. G3-4) were also not significantly associated with cancer-specific survival (p = 0.49 and p = 0.15, respectively).

## Discussion

The role of human high-risk papillomavirus infection as a causative agent in the development of penile squamous cell carcinoma is well established [20]. However, the impact of HPV-related tumourigenesis on tumour morphology as well as aggressiveness is still subject to research and has led to contradictory results [15-17]. Moreover, it is still not clear to which extent immunostaining for p16^INK4a^ might be regarded as a surrogate marker for HPV infection, since site-specific differences in sensitivity and specificity have been reported [10,21-23]. The observed variety of staining patterns for p16^INK4a^ further complicates histologic evaluation [24,25]. Therefore, the aim of this study was to evaluate p16^INK4a^ staining patterns and results from HPV DNA subtyping with histopathological and clinical characteristics in a cohort of 58 patients with penile squamous cell carcinoma (pSCC). Among the study cohort, 91% of pSCCs were classified as conventional-type (keratinizing pSCC) because various degrees of keratinization could be observed in these cases. This is a higher proportion than has been described in earlier studies, while the rate of basaloid-type tumours was comparable to data from literature [11,16,26]. HPV DNA (of any subtype) was detected in 31% of all cases, with HR-HPV DNA (HPV-16 and HPV-45) being present in 28% of conventional-type and 40% of basaloid-type carcinomas. Thus, despite a specificity of 95% and a positive predictive value of 40%, the resulting sensitivity of basaloid tumour differentiation for the presence of HR-HPV DNA was only 11%. Due to the significant proportion of conventional-type (keratinizing) tumours that turned out HPV-positive in PCR-based assays, our results do not support the correlation between presence of HR-HPV DNA and basaloid tumour subtype that has been previously described by other authors. This might be due to the fact that distinguishing one histological type from another can be challenging, and overlapping differentiation patterns as well as intratumoural heterogeneity exist [27]. Therefore, we would not recommend to rely solely on basaloid tumour subtype when assessing hints for HPV-driven tumourigenesis as proposed by some authors but rather use a combination of criteria as proposed in a 2014 study by Chaux et al. [28,29]. Positive immunostaining for p16^INK4a^, a tumour suppressor protein that is regarded as a surrogate marker for HPV-associated tumours in other organs [7,30], was observed in 59% of specimens. There was a wide variety from scattered-focal over confluent-intense to almost exclusively nuclear staining patterns. P16^INK4a^ immunostaining correlated significantly with the presence of HR-HPV DNA with good sensitivity (100%), but lacked specificity (60%) when all staining patterns were considered (PPV, 53%). Considering only those specimens with intense nuclear positivity for p16^INK4a^ in all tumour cells improved the specificity for the presence of HR-HPV DNA to 85% (PPV, 75%). P16^INK4a^ was present in all HPV-positive cases; however, lack of p16^INK4a^ immunostaining in HPV-associated tumours due to loss of heterozygosity near the *CDKN2A* locus and/or hypermethylation of the *CDKN2A* promoter has been described previously [31]. It is therefore well conceivable that these genetic aberrations accumulate stepwise during tumour progression, and could not be observed here due to the relatively large proportion of early/noninvasive HPV-positive tumours in our study. Also, it has to be stated that we did not examine p16^INK4a^ expression in corresponding metastases, but a 2014 study showed identical immunohistochemical or HPV in situ hybridization profiles between primary pSCCs and their corresponding metastases [32]. However, the question remains if there is a prognostic value of HR-HPV-driven tumourigenesis in pSCC at all. To address this, we investigated whether presence of HR-HPV DNA was associated with tumour aggressiveness or cancer-specific survival in pSCC. We found that HR-HPV as well as p16^INK4a^ positivity was significantly associated with non-invasive tumour growth (pTis/pTa stage). This finding is in contrast to a proposed proinvasive role for HPV oncoproteins that has been recently described in head and neck as well as cervical cancer, but is supported by data for penile cancer published by other authors [13,16,33-35]. Our findings therefore add to the growing evidence that there are site-specific differences in the role of HPV regarding the gain of an invasive phenotype. These differences may be linked to interaction of HPV-derived oncoproteins with β-integrin localization and signalling [36]. For nodal or distant metastasis, there was no significant correlation with presence of HR-HPV DNA, p16^INK4a^ staining or histologic grade in our sample set; similar results have been previously described [37]. Furthermore, we detected no significant differences in cancer-specific survival with regard to histologic differentiation grade, or p16^INK4a^ positivity. This is in line with some previous studies that failed to confirm a proposed association between histopathologic grade and lymph node metastasis or overall survival in pSCC, while it has to be stated as a clear limitation of our study that follow-up data was only obtainable for 35 patients (60.3%) [38-40]. Considering the substantial interobserver variability that has been described for histopathologic grading in pSCC (between 59-87% with ĸ = 0.38-0.69 [41]), we think that the current histopathologic grading is of limited value due to an obvious lack of prognostic relevance; this conclusion is also supported by other authors [42]. For the presence of HR-HPV we observed a trend towards better prognosis that failed to reach statistical significance; further investigations in larger cohorts might therefor be indicated.

## Conclusion

In this study, we evaluate histologic subtyping as well as p16^INK4a^ immunostaining for detection of HR-HPV DNA and propose that ubiquitous nuclear expression of p16^INK4a^ is a strong predictor for the presence of HR-HPV in penile squamous cell carcinoma. We further show that, in contrast to histologic grading, presence of HR-HPV DNA is associated with noninvasive growth of the tumour. However, since there was no significant impact of HPV-driven tumourigenesis on metastasis and cancer-specific survival in pSCC, routine p16^INK4a^ immunostaining or HPV genotyping in pSCC cannot be recommended.

## Abbreviations

HNSCCs: Squamous cell carcinomas of the head and neck region
HR/LR-HPV: High-risk/ low-risk human papillomavirus
PCR: Polymerase chain reaction
PPV: Positive predictive value
pSCC: penile squamous cell carcinoma
UICC: Union internationale contre le cancer
